# The molecular evolution of vertebrate organs

**DOI:** 10.1038/s41559-026-03003-7

**Published:** 2026-02-25

**Authors:** Margarida Cardoso-Moreira

**Affiliations:** Evolutionary developmental biology laboratory, Francis Crick Institute, 1 Midland Rd, London NW1 1AT, UK

## Abstract

The ecological and evolutionary success of vertebrates stems in large part from their remarkably diverse organs. Understanding how this organ diversity has arisen has been a long-standing goal. What molecular and developmental changes are responsible for the origin and diversification of vertebrate organs, and what evolutionary forces drove these changes? This Review discusses major leaps in our understanding of the molecular evolution of vertebrate organs made possible by technological advances—i.e., whole-genome sequencing, functional and single-cell genomics, and genome editing tools. I summarize how organs and cell types evolve at different rates, and the distinct contributions that some types of molecular change make to organ evolution. Finally, I discuss how new, complex traits evolve—from cells to tissues and organs—and how these innovations have fuelled the diversification of vertebrates.

Vertebrate organs are remarkably diverse. Their diversification powered vertebrates’ transition from water to land and their adaptation to most of the planet’s environments^1^. Vertebrates share a common set of internal organs—including the brain, heart, kidney, liver, and gonads— that was inherited from their chordate ancestors more than 500 million years ago^1^. While shared by all vertebrates, their forms and functions vary widely. For example, vertebrate brains vary across species in their main brain regions, the size of the areas allocated to sensory modalities, and in the organisation of neurons and neural circuits (Box 1, Fig. 1)^1^. Vertebrate’s hearts vary in the number of chambers, valves, and regenerative capacity^2^ (Fig. 1). Other shared organs are similarly diverse. Additionally, throughout vertebrate evolution, new organs and tissues have emerged, including the spleen^3^, stomach^4^, lung^5^, and placenta^6,7^, and as new organs emerged, others were lost (Fig. 1).

How did this organ diversity arise? What are the molecular and developmental changes that led to the origin and diversification of vertebrate organs? And what were the evolutionary forces driving their evolution? These are long-standing questions that are now addressable thanks to new technologies. The combination of whole-genome sequencing, functional and single-cell genomics, and genome editing tools—technologies that can, in principle, be applied to any species, cell, and organ—is transforming and expanding our understanding of the evolution of vertebrate organs. This review discusses the growing number of functional genomics studies (e.g., genomics, transcriptomics, epigenomics) that are revealing the molecular, cellular, and evolutionary mechanisms underlying the origin and diversification of vertebrate organs. Although the review focuses on vertebrates, the principles and mechanisms identified are relevant to organ evolution in other animals and are likely to be relevant to multicellular organisms as a whole.

## Molecular basis of organ evolution

A great diversity of molecular changes underpins the phenotypic diversification of vertebrate organs. These include mutations (at the transcriptional and post-transcriptional levels) that alter when, where, and how much of a protein is produced, mutations that change protein sequences (including creating novel isoforms), and structural mutations leading to gene gains and losses. While all these changes contribute to the evolution of vertebrate organs, questions remain about whether some types of change make distinct contributions to phenotypic evolution. For example, are gene expression changes the most common driver of phenotypic differences? If so, is this because mutations in regulatory elements tend to be less pleiotropic^8–12^? Are changes in morphology more likely driven by changes in gene expression, while changes in physiology by changes in protein sequences and gene duplication^8,9,13^? Are gene duplications, especially those from whole genome duplications, critical for vertebrate innovations^14–16^? Can transposable elements rapidly rewire gene regulatory networks and thus drive major phenotypic changes^17–19^? These questions remain hotly debated because identifying the genetic basis of organ evolution remains challenging^12,20^. Most traits emerged from many genetic changes, often a combination of different mutation types, dozens to hundreds of millions of years ago^12,20^. Still, omics technologies are expanding our view of the molecular changes driving organ evolution, their relative prevalence, their co-occurrence, and how they differ across organs and cells.

### Organs and cells evolve at different rates

In the second decade of this century, it became both technically and economically feasible to apply genome sequencing and functional genomics widely across vertebrates. These technologies allowed cataloguing molecular differences across organs and species in terms of gene expression^21–26^, alternative splicing^27–30^, small^31^ and long non-coding RNAs^32,33^, regulatory elements^34–36^, epigenetic marks^37–40^, gene evolutionary ages^24,41^, rates of translation^42^, and coding sequence evolution^24,43^. When these technologies were applied simultaneously to multiple organs across multiple species, the molecular data could be directly compared and it showed how quickly (or slowly) different vertebrate organs have evolved. Perhaps surprisingly, given the anatomical and functional diversification of vertebrate brains and their regions^1^ (Box 1), studies consistently identified the brain as the slowest-evolving organ. In contrast, the testis is the fastest evolving. These differences in the rates of molecular evolution between organs are consistent across evolutionary scales, from major vertebrate lineages^25,26,32^ to mammals^22,24,32,33,42^, primates^21,22^, and the recent diversification of cichlid fishes^23^.

Our understanding of why the testis evolves faster than all other organs had to wait for single-cell technologies to enable cross-species comparisons at the level of individual cell types. We expect differences in evolutionary rates among organs to result from differences in the evolutionary rates of their constituent cell types. However, it is unknown how much variation in evolutionary rates exists among the cell types of an organ, and hence whether the rates of molecular evolution observed at the organ level (using bulk RNA sequencing) reflect those of most or only a subset of cell types. In the case of the testis, comparative single-cell studies revealed that the fast molecular evolution observed at the organ level is driven specifically by late-spermatogenic cells^44–47^. These cells experience fewer selective constraints than other cells and increased levels of positive selection^47,48^. Could this fast evolution also extend to female germ cells? Organ-level studies found that the ovary evolves considerably slower than the testis^22,24^. However, this could reflect the fact that late-stage female germ cells make up a small fraction of the adult ovary, while late-stage male germ cells make up the bulk of the adult testis. In agreement with this idea, a recent single-cell study showed that late-stage female germ cells are also fast-evolving^48^.

In contrast, in the brain, most cell types appear to be evolving slowly. The mammalian brain is slow-evolving^22,24^ and comparative single-cell studies found that astrocytes and most neurons are correspondingly slow-evolving cells^49,50^. In the mammalian cerebellum, the fastest-evolving cells are microglia^50^, a resident macrophage, consistent with immune cells being generally fast-evolving cells^49^. What is notable is that these immune cells of the brain are the slowest-evolving immune cells in the body^50,51^, matching the slow evolution of their neighbouring cells. It is an intriguing question whether cells shared across organs, like fibroblasts or tissue-resident immune cells, evolve at rates that reflect their organ-specific cell neighbours, and if so, why. With cell atlases becoming available for multiple organs across multiple vertebrates^47,52–57^, we should soon have a comparison of the rates of molecular evolution across all vertebrate cell types. Early studies suggest that the same cell types may mediate most adaptation across species. For example, in the intestine, the epithelial cells that specialise in nutrient absorption (enterocytes) are the fastest evolving intestinal cells in both primates^56^ and chiclids^55^, and are suggested to be key mediators of these species’ unique dietary adaptations.

### Are some types of molecular change more prevalent in specific organs?

The differences in the rate of molecular evolution across organs are consistent across types of molecular change, including coding and non-coding gene expression^22–24,26,32,33^, translation^42^, alternative splicing^29^, sequence evolution^24^, and gene evolutionary age^24^. This means that when organs evolve faster, they do so through multiple types of molecular change in parallel^21,24,26,35^. The mammalian liver provides a good example: recently evolved enhancers that likely drive new expression patterns are over-represented near positively selected genes^35^.

However, while it is true that the organs that differ the most between species in expression levels are also the ones expressing more species-specific genes and genes under positive selection^21,24^, some types of molecular change may be more prevalent in specific organs or cell types. A case in point is alternative splicing, which plays a more prominent role in the evolution of the brain and heart than of other organs^27–29^, probably because alternative splicing is more extensive in neurons and cardiomyocytes (and other muscle cells) than in other cell types^58,59^. Another example is gene expression changes leading to species differences in the timing of developmental events (heterochrony), which are most common in the evolution of the ovary and testis^24,45,60^.

Recent years have seen a burst of studies on the roles of transposable elements in vertebrate evolution. Transposable elements contribute to organ evolution by adding new exons and genes to genomes^19,61–63^, like syncytins, the proteins that mediate cell-cell fusion in mammalian placentas^61,64^. They also contribute to organ evolution by changing gene regulation^17–19,63,65–68^. Because transposable elements can act as regulatory elements for multiple genes, they could, in principle, drive evolutionary change by ‘rewiring’ entire gene networks^17–19^. A current challenge for the field is to go beyond correlations and demonstrate that transposable element-mediated gene network rewiring has contributed to organ evolution. Nevertheless, studies of the immune system^65^ and pregnancy^66–69^ suggest this could be the case. Transposable elements contribute disproportionately to the evolution of fast-evolving organs or cells, namely the testis^70^, blood and immune cells^65,70,71^, and the placenta^64,66–72^, suggesting they play key roles in adaptation.

### Whole genome duplications and vertebrate innovations

**Figure 1:**
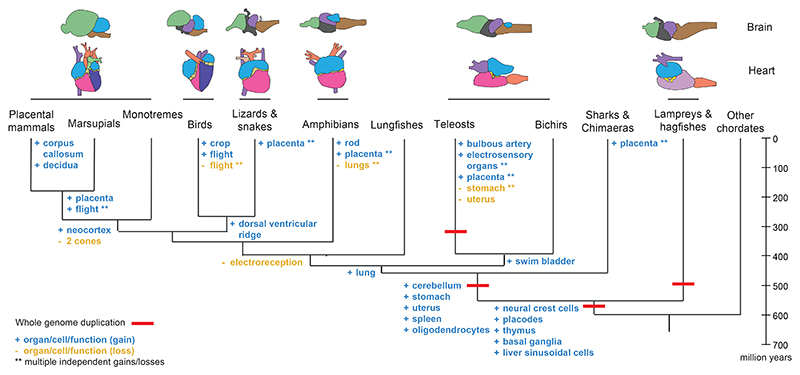
Diversity of brains and hearts, and examples of gains and losses of organs and cell types, throughout vertebrate evolution.

One type of molecular change has long loomed large over vertebrate evolution: whole-genome duplication^14,15^. In 1970, Ohno hypothesised that two rounds of whole-genome duplication occurred early in vertebrate evolution, fuelling the emergence of innovations and vertebrate diversification^14^. It took time to confirm both rounds of whole-genome duplication, and only recently have they been completely reconstructed^73–76^. The first whole-genome duplication occurred early in vertebrate evolution (~530 mya), in the common ancestor of jawed and jawless vertebrates (hagfish and lampreys). After these two lineages split, two additional rounds of genome duplication occurred around 500 mya: one each in the ancestors of jawed and jawless vertebrates^73–76^ (Fig. 1). Further rounds of genome duplication occurred among jawed vertebrates, most frequently in fish^77^, including a third genome duplication in the ancestor of teleost fish ~320 Mya^77^ and in the non-teleosts paddlefish^5^ and sterlet sturgeon^5,78^, and a fourth genome duplication in salmonids and cypriniformes^77^. Consistent with Ohno’s hypothesis, the genes retained after the whole-genome duplication events (which we now call “ohnologs”) are enriched for transcriptional and developmental regulators^15,73–76^, genes that could underlie vertebrate innovations and morphological diversification.

Despite these compelling observations, causal links between ohnologs, vertebrate innovations, and morphological diversification remain elusive^15,79^. Whole-genome duplications are associated with the radiations of vertebrates and teleosts but they also occurred in lineages that did not experience radiations^77^. When the teleost radiation was re-examined in light of the fossil record, no correlation was found between the whole-genome duplication and the teleost morphological diversification^80^. Establishing a direct link between genome duplications and diversification events is further complicated by the possibility of a time lag between them. The functional impacts of whole-genome duplications may arise only gradually, tens of millions of years after the duplication events^81,82^. The link between ohnologs and the emergence of vertebrate innovations is also tenuous. Vertebrate innovations like neural crest cells and the vertebrate brain and sensory organs originated before the first round of whole-genome duplication early in vertebrate evolution^15,16,76,79^ and, therefore, are not directly linked to ohnologs.

Like other duplicates, with time, ohnologs functionally diverge from each other. This can occur through subfunctionalisation, when ancestral functions are split between ohnologs (division of labour), or through neofunctionalisation, when one or both ohnologs acquire a new function^15,76,79,82–85^. Both subfunctionalisation and neofunctionalisation can occur through changes in coding sequences, changes in gene regulation, or a mix of both. What sets ohnologs apart from other duplicates is that whole-genome duplication is the only mechanism by which genes that are dosage-sensitive or part of macromolecular complexes or pathways can duplicate without deleterious consequences^15,84,86^. Ohnologs are therefore a distinct set from genes arising from small-scale duplications and are uniquely enriched in regulatory and developmental genes^87,88^.

Consistent with these characteristics, ohnologs play key roles in the development of vertebrate-specific traits, including the origin of new cell types^83^. For example, the liver’s sinusoidal endothelial cells are a vertebrate novelty which depend on ohnologs for their developmental specification and function^53^. Other liver functions, such as a vertebrate-specific pathway for bile production, also rely on ohnologs^53^. Ohnologs are also enriched among genes expressed in the lungs of most vertebrates^57^ and have played a disproportional role in the evolution of brain cell types throughout vertebrate history^83^. In teleosts, ohnologs from the third round of whole-genome duplication are necessary for the development of the electrosensory system of some fish^85^ and the bulbous arterious^85,89^, a specialisation of the teleost heart.

## Evolutionary forces driving organ evolution

Cataloguing molecular differences between species tells us about the end products of evolution, but not which microevolutionary processes (e.g. population genetics) were responsible^90^. While neutral processes^91,92^—mutation, genetic drift, recombination, migration—shape important aspects of genome evolution, adaptive processes (positive selection) are assumed to be key for phenotypic evolution^12,92^. This expectation is particularly strong for the evolution of novel functions and structures. In contrast, trait loss (including organ loss) could be driven by positive selection, a relaxation of selective constraints, or a mixture of both^93^. Although the dominant view is that adaptive processes drive most phenotypic organ evolution^12,20^, this position has been challenged on theoretical and empirical grounds^90,94^. The evolution of some traits, including complex traits like new cell types and tissues^94^, may result from an interplay of neutral and adaptive processes.

### Multiple forces underlie organ evolution

Identifying the evolutionary forces driving organ evolution remains one of the field’s most challenging and sought-after goals. Purifying (stabilising/negative) selection dominates the evolution of functional genomic elements and can be readily detected through the evolutionary conservation of coding sequences, regulatory elements, and expression levels^21,22,43,95^. Detecting positive selection is more challenging^96,97^. Across birds and mammals, the strongest population genetic and genomic signals of adaptation are associated with genes involved in immunity, reproduction, diet and response to xenobiotics^96,98–101^. However, it is much easier to detect positive selection in genes that are common targets of adaptation than in genes underlying lineage-specific adaptation^96^. Fortunately, the rise in sequencing capacity means hundreds of vertebrate genomes will soon be combined with population resequencing data, greatly increasing the power to detect positive selection^96^. Promising approaches that combine population and quantitative genetics are also being developed^97^.

Different evolutionary forces and differences in the strength of these forces explain why vertebrate organs and cells evolve at different rates. While purifying selection is pervasive, its strength differs across organs^21–24,102,103^. Genes expressed in the brain experience the strongest selection both at the sequence and expression levels and, consequently, differ the least across species^21–24,102^. In contrast, genes expressed in the testis (specifically in late-spermatogenic cells) experience weaker purifying selection and differ the most across species^21–24,102,103^. Positive selection amplifies these differences between organs. Organs like the liver, which mediate interactions with the environment (e.g. diet, toxins, communication through pheromones), are often targets of adaptation and evolve rapidly^23,24,102,104^. Sperm competition, sexual conflict and meiotic drive have all been proposed to explain the rapid evolution of vertebrate testes in addition to weaker purifying selection^21,22,47,102^.

An area of increasing interest is the evolution of sex differences (Box 2). These include gene expression differences between male and female organs, which evolve particularly fast. Identifying the evolutionary forces driving these differences is difficult because detecting positive selection using expression levels or regulatory elements remains challenging^95,102,105,106^. While some studies have proposed that genetic drift^107^ and reduced purifying selection^108^ drive most organ sex-biased expression, others invoked natural^104^ and sexual ^104,109^ selection as the main drivers.

### Interplay between evolution and development

As evolution and embryology rose as disciplines in the 19th century, a link between them emerged. Karl von Baer, a founding father of embryology and Darwin’s contemporary, noted that early embryos of different species are morphologically similar but that as development progresses, species differences emerge, and embryos become increasingly distinct from each other^110^. Von Baer’s observations influenced Darwin^111^, but while we have long known that evolution and development are intertwined, we don’t fully understand why that is^110,112,113^.

Organ development starts after the most conserved stage of embryonic development (the phylotypic period^24,29,33^). As organogenesis proceeds, organs become more specialised, with the cell types that make up the adult organs becoming progressively specified. As organ development progresses, what von Baer described for the embryo at the morphological level also occurs for organs at the molecular level. The earliest stages of organ development are the most conserved, with species differences increasing as development progress^24,29,33,114^. Two non-mutually exclusive hypotheses can explain this pattern^112^. One proposes that functional constraints are higher earlier than later in development, leading to stronger purifying selection against mutations affecting early development, resulting in higher conservation early on. The other hypothesis proposes that adaptation drives most species differences and that adaptation occurs more often late in development when environmental pressures are stronger. Adaptations of mature organ functions would be encoded in changes to later developmental programs when these functions are specified. Molecular tests of both hypotheses using mammalian organs showed that both stronger purifying early in development and increased adaptation later in development contribute to the “von Baerian” divergence.

**Figure 2:**
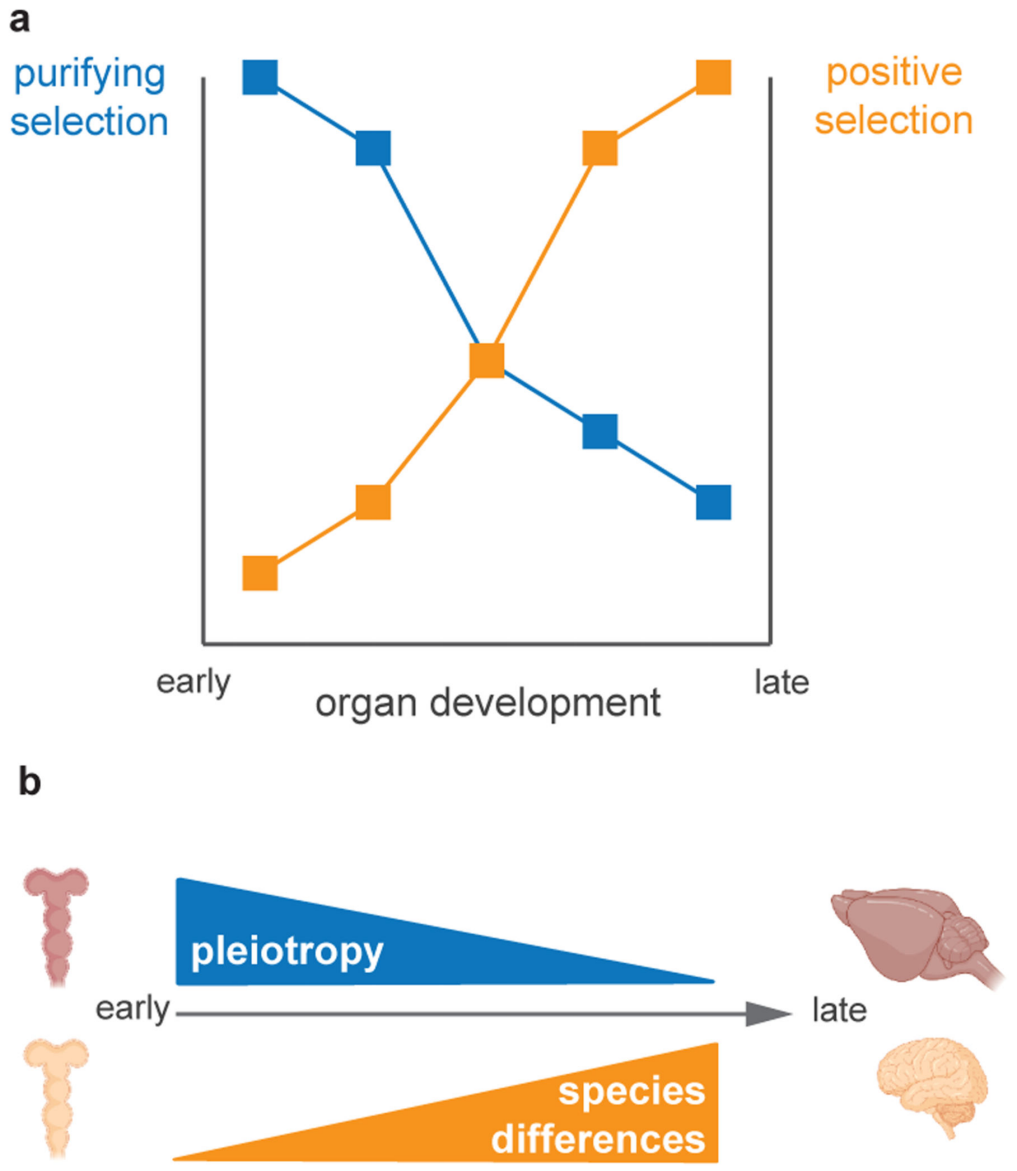
(a) As development progresses, purifying selection decreases while positive selection increases. (b) As development progresses, the pleiotropy of the genes employed decreases and differences between species (molecular, morphological, etc) increase.

One factor may account for both stronger functional constraints early in development and increased adaptation later: the changing pleiotropy of the genes employed during organ development ^24,110,115,116^. Pleiotropy refers to the number of traits a gene or a mutation impact^24,110,115,116^ and it determines the types of mutation permissible under selection^8–10^. The more pleiotropic a gene or a mutation is, the stronger the purifying selection; conversely, mutations with few or no pleiotropic effects are more likely to underlie adaptation ^8–10^. Evidence from vertebrate embryos and mammalian organs supports a decrease in the pleiotropy of the genes expressed during development: gene expression becomes more temporally and spatially restricted with time^24,29,33,116^ (Fig. 2B). These findings likely reflect the fact that organ development proceeds from the more general to the more specialised and suggest that in a developmental system where pleiotropy decreases over time, a “von Baerian” relationship between evolution and development emerges (mammalian teeth may be an exception^117^). Indeed, single-cell studies of cell differentiation in the mammalian cerebellum^50,54^, testis^47,48^, ovary^48^, and intestine^55,118^ found that as cells differentiate, species differences in gene expression and regulation increase. Because the evolution of developmental programs underlies species differences, an increasing number of studies are combining evolution with development to understand the diversification of vertebrate organs.

## The origin of evolutionary novelties

In recent years, whole-genome sequencing, functional and single-cell genomics, and genome editing have been applied to numerous vertebrate species. These approaches are uncovering common trends in organ evolution. In addition, they are providing much-needed insights into how evolutionary novelties originate. Recent studies have begun to identify the molecular changes and evolutionary forces that drive the evolution of new forms and functions, the emergence of new cell types, and even the origin of new organs.

### Evolution of new forms and functions

There are many open questions on the genetics of adaptation^12,119^. Do changes in morphology and physiology arise from the accumulation of many genetic changes of small effect, or a few changes of large effect? Are these genetic changes mainly altering gene structure (amino acid substitutions or gene duplications) or gene regulation? Are there classes of genes more likely to drive phenotypic evolution, and if so, does it depend on the type of phenotype? Do genetic linkage and pleiotropy facilitate or hinder phenotypic evolution? Answers to these and related questions are emerging from multiple study systems, most notably from studies of traits that have evolved convergently in multiple lineages.

Sticklebacks are marine fish that have recently adapted, multiple times, to life in freshwater habitats. Many morphological, physiological, and behavioural traits have evolved convergently in these freshwater populations, and their combined study has answered key questions on the genetics of adaptation^12,119^. In sticklebacks, most new phenotypes appear to have evolved through a few mutations of large effect and many more of smaller effect (in agreement with theoretical work^120^). Adaptation has typically occurred through changes in gene regulation, predominantly of developmental genes. In cases of repeated evolution, half the time the same genes are involved, although the likelihood of gene reuse decreases with increasing evolutionary divergence^121^.

A notable example of phenotypic convergence in mammals is the evolution of flight. Lateral flight membranes (patagia) have evolved independently at least seven times in mammals creating gliding species. The evolution of the patagia shares features observed in the evolution of phenotypes in sticklebacks: it occurred via molecular changes in the regulatory elements of pleiotropic developmental regulators, and the same genes were reused during the evolution of the convergent trait^122,123^. Pleiotropy may have facilitated the evolution of the patagia because the deployment of an existing patterning mechanism in a new context may have facilitated the functional integration of the novel structure^122^. Similar principles underlie the one-time evolution of powered flight in mammals. The evolution of the bat wing occurred partly through changes in the regulation of developmental genes, leading to the redeployment of an existing transcriptional program at a different anatomical location^124^. Given the preponderance of changes in regulatory regions in the evolution of flight in mammals, it is perhaps unsurprising that the repeated, independent losses of flight in birds also mainly resulted from changes in regulatory elements^125^.

While the frequent involvement of pleiotropic developmental regulators in the evolution of limb and skeletal features is notable^122–126^, these are very specific phenotypic traits. Coding sequence changes and duplications and losses of different types of genes have undoubtedly also played key roles in the evolution of organ phenotypes. As new technologies are widely deployed across the vertebrate phylogeny, new observations, trends, and principles await discovery.

### The evolution of new cell types

Vertebrates are made of several hundred different cell types, over a quarter of which are neurons^127^. Many cell types were inherited from chordate ancestors, but several others emerged during vertebrate evolution. The oldest vertebrate-specific cells emerged ~550 million years ago, and the youngest cell type currently known is only ~20,000 years old^128^.

The origin of vertebrates is linked to the evolution of two embryonic cell populations: the neural crest and cranial placodes^1,129–131^. These two vertebrate innovations give rise to cell types that reshaped the vertebrate head, heart, and sensory systems. Neural crest cells differentiate into many different cell types, including those that make bone, cartilage, smooth muscle, and peripheral nerves^79,130,131^. Cranial placodes form many of vertebrates’ sensory structures, including the inner ear, nose, lens, and neurons of cranial sensory ganglia^132^. While neural crest cells and cranial placodes are defining features of vertebrates, their evolutionary precursors can be found in invertebrate chordates^130–132^. The neural crest and cranial placodes evolved in a stepwise manner from precursors that possessed some (but not all) of their morphological and molecular features, through the co-option of existing genes and gene networks^79,131,132^. The neural crest became a new developmental path to specify existing cell types. Several cell types still show their dual origin (from neural crest and another source), some are now only specified by the neural crest (but existed prior to the neural crest emergence), and others are new cell types altogether^127,131,133^. For example, several sensory neurons and other cell types crucial to the transition from water to land are neural crest-derived^132^, as are lipochondrocytes, lipid-filled cells that make a new type of cartilage resistant to deformation and tear, that are a mammalian innovation^134^.

Several models have been proposed to explain the evolution of new cell types^133,135–138^ (Box 3). While distinct, they assume that cell identities result from a unique combination of cell type-defining transcription factors, referred to as terminal selectors^139^, or in conjunction with other genes, core regulatory complexes^135^, or character identity networks^140^. The best understood is the sister cell type model, whereby two different cell types originate from a single multifunctional cell. Neurons are thought to mostly evolve this way^133,135,137^. For instance, retinal photoreceptors and bipolar cells likely originated from an ancestral multifunctional cell^133,141^. The same principle applies to the evolution of photoreceptors. The ancestor of vertebrates had one type of rod and four types of cones, and new photoreceptors were gained and lost throughout vertebrate evolution^142,143^. For example, amphibians evolved a new type of rod to detect colour in dim light, while mammals lost two types of cones as they adapted to nocturnality^142,143^. Oligodendrocytes are yet another example of a sister cell type. They emerged from an ancestral multifunctional glial cell with properties of both astrocytes and oligodendrocytes^144,145^ in the ancestor of jawed vertebrates, introducing myelination to the central nervous system.

The evolution of new cell types can lead to the evolution of new tissues. For example, the emergence of the cerebellum required the evolution of Purkinje cells, a neuronal type unique to this brain region^1^. Similarly, the origin of the maternal decidua, which is key for pregnancy in placental mammals, depended on the evolution of decidual cells. These cells evolved in the ancestor of placental mammals as a sister cell type to endometrial fibroblasts. Decidual cells evolved in a stepwise manner, initially acquiring immunomodulatory characteristics and later, in some lineages, endocrine functions^146^. Multiple molecular changes drove the evolution of the decidual cell, including amino acid changes in key transcription factors^147^ and convergent gene expression changes mediated by transposable elements^148^.

### Gains and losses of organs

Several organs emerged throughout vertebrate evolution (Fig. 1). Some systems — the digestive, reproductive, sensory, and immune systems — are hotspots for organ innovation. Examples include the stomach in jawed vertebrates^4^ and the crop in birds^149^ (digestive); the uterus in jawed vertebrates^150^ and over 100 independent origins of a placenta^6,7^ in different vertebrate lineages (reproductive); the thymus in the vertebrate ancestor^3^ and the spleen in jawed vertebrates^3^ (immune); multiple independent origins of electrosensory organs in fishes^85^ (sensory); plus the evolution of the lung in the ancestor of bony fishes^5^ and the evolution of the swim bladder (from the lung) in ray-finned fishes^5^. The list goes on.

Because organs don’t work in isolation, when a new organ emerges, other organs may need to co-evolve with it. For example, the evolution of a placenta requires changes to the mother’s immune system, metabolism, and cardiovascular function^7^. This principle applies to existing organs as well, and major ecological shifts, like vertebrates’ transition from water to land, required adaptations across multiple organs^5^.

Despite the many examples, we know little about how new organs originate. One possibility is that an ancestral multifunctional organ could split its functions between two newly evolved structures, which then specialise further. This might explain how some digestive organs evolved. Organs could also evolve by the co-option, or repurposing, of an existing structure (also called exaptation). For example, the swim bladder was co-opted multiple times independently as a sound-producing organ in teleost fish^151^. Alternatively, new organs could emerge through successive rounds of cell type innovation^94^. The sister cell type model (Box 3) makes this transition easier because sister cell types are bound together developmentally, spatially, and temporally, which could facilitate the evolution of a new structure with a new function. Eyes and parts of the brain may have emerged this way (Box 1)^133,135,137^. However, while new cell types have played a crucial role in organ evolution, they may not be essential. Novel interactions between existing cell populations and functional diversification of existing cell types could also lead to the evolution of new organs^6^. Indeed, it is an open and important question how often the evolution of new organs is a consequence of the evolution of new cell types and how often it occurs through the repurposing of existing cell types.

The history of vertebrates is also marked by organ loss (Fig. 1). These include multiple independent losses of the stomach in fish lineages^4,152^, the loss of the uterus in teleost fishes^150^, and the independent losses of the lung in many salamanders^153^. The stomach evolved in the ancestor of jawed vertebrates as a gut pouch that secretes hydrochloric acid and pepsin, improving digestion and protecting from pathogens^4,152^. Stomachs have been lost more than 15 times independently^4,152^. Loss of the stomach among fish lineages is associated with loss of similar genes across species. Because these genes are essential for stomach function^152,154^ it is very unlikely that a stomach loss can be reversed^4,152^, an example of Dollo’s law^155^: the idea that complex traits that have been lost in evolution cannot be regained. Still, organs are rarely lost without a trace. Many leave vestigial structures, likely because they play a role during early development, that are a useful record of vertebrate history^93,115,153^.

## Conclusion

The study of organ evolution is part of larger research programs currently in a golden period: the genetics of adaptation and the evolution of new and complex traits^156^. The explosion of omics data and our ability to study organs at the cellular level in any species of our choice is fuelling much of this renaissance. These advances allow the study of complex traits in non-model species, radically forwarding our understanding of how organs originate and how they diversify in form and function across species. But several challenges remain. Reconstructing the evolution of vertebrate organs will require bridging between micro- and macro-evolutionary processes. It will also require a closer integration between evolution and development, which is already underway^24,104,122–124,146,157,158^. In the coming years, we will see increased attention paid to how mechanical forces influence the evolution of new traits^159,160^, and more studies will combine population genetics with developmental biology^55,119,126,128^. We will also see the rise of new technologies and statistical tools^161,162^, and with them, our ability to reconstruct and understand the evolution of vertebrates.

## Box 1 The evolution of the vertebrate brain

Vertebrates are characterised by a distinctive head housing a large, complex brain with specialised regions^1^. Vertebrate brains share the same basic architecture with three main divisions: forebrain, midbrain, and hindbrain^1^. These divisions give rise to different brain regions with different circuits and neuron types. Several new brain structures emerged during vertebrate evolution, including the cerebellum in jawed vertebrates, the neocortex in mammals, and the corpus callosum in placental mammals, while some brain abilities were lost, like the electroreception of amniotes with their transition to land^1,163,164^.

There is extensive variation among vertebrates across all levels, from brain regions to circuits and neuron types^1^. For example, the amniote pallium includes the neocortex of mammals and the dorsal ventricular ridge of birds and other reptiles, structures that mediate the complex cognitive abilities of these species^1,165–167^. It is heavily debated whether the neocortex and the dorsal ventricular ridge are homologous or evolved convergently. Evidence from neural circuitry, physiology, and development supports differing conclusions^165–167^. Recent single-cell studies favour the convergent evolution of the mammalian neocortex and the avian pallium and show that brain regions and circuits are mosaics of deeply conserved and newly evolved neurons^157,158,168–171^.

New brain regions can evolve through the duplication of existing regions. This can occur through a coordinated increase in cell numbers, followed by the functional divergence of cell types and anatomical regionalisation. Examples include the basal ganglia of vertebrates^137,172^ and the cerebellum of jawed vertebrates^164^. The cerebellum is made of functional units that have duplicated and diverged several times: cartilaginous fishes and amphibians have one unit pair, reptiles have two, and mammals have three. New regions may also evolve by splitting a multifunctional region into more specialised ones, or they may evolve *de novo* from the evolution of new cell types and/or by a novel combination of cell types^137,170^.

Cell type diversification has shaped vertebrate brain evolution. For example, mammals have hundreds of telencephalic excitatory neurons, whereas only a handful have been described in the jawless vertebrate lamprey^145^. Rates of cell type evolution differ among major neuronal classes. Among amniotes, inhibitory neurons have conserved identities across species. Excitatory neurons, however, evolve more rapidly, with many novel excitatory neurons emerging during amniote evolution^157,158,168,169,171^. Changes in function and behaviour do not, however, always require the evolution of new cell types. Changes in the abundance of neurons, their locations and projections, and the genes they express, likely explain many of the functional and behavioural differences found across vertebrates.

## Box 2 The evolution of sex differences

Sex differences are widespread across vertebrate organs. Despite most early developmental processes being highly conserved, sex determination and sex differentiation mechanisms are highly variable across species^173^. For instance, vertebrates differ in having genetic or environmental sex-determination mechanisms and in the cell types and genes that initiate the sex-determination cascades^173^. The mechanisms of somatic sex differentiation that occur later in development also differ across species. For example, in mammals, sex differentiation is mainly driven by hormones, while in birds each cell carries a sex identity defined by its sex chromosomes and sex differentiation is primarily cell-autonomous^173,174^.

Sexual dimorphisms are often studied through the proxy of sex-biased expression. Across mammals, birds and fishes, sex-biased expression evolves quickly^104,107,108,175,176^. Levels of sex-biased expression differ across species and organs, as does the identity of the sex-biased genes^104,107,108,175,176^. Sex-biased expression tends to be organ-specific, and single-cell studies suggest this is because sex differences are restricted to specific cell-types^104,176,177^. Interestingly, at least in mammals, the cell types that show sex differences are the same across species, even if the sets of sex-biased genes are different^104^.

Why do organ sex differences evolve so fast? While genetic drift plays a role^107,108^, it is likely because of the combination of strong sexual and natural selection^104,109^ and a simple genetic basis. In mammals, sex differences are mostly under hormonal control, and genes can gain or lose sex-biased expression through relatively simple changes in binding sites for hormone-responsive transcription factors^104^. It remains an open question whether the same applies to vertebrates like birds, where sex identity is primarily cell-autonomous.

## Box 3 Models of new cell type evolution

Four models have been proposed to explain the evolution of new cell types. The most studied is the **sister cell type** model, which can explain the evolution of neurons and neuronal circuits, as well as many non-neuronal cells^133,135^. In this model, multifunctional cells give rise to new cell types by segregating their functions among sister cell types (division of labour). This segregation is achieved through the selective loss of expression, in each sister cell type, of effector genes that were active in the ancestral cell. This could be achieved through the partitioning among the sister cell types of the ancestral cell type-defining transcription factors (also called terminal selectors^139^, or in combination with other genes, core regulatory complexes^135^, or character identity networks^140^). The new sister cell types can further diverge and gain new functions by co-opting existing gene networks or recruiting newly evolved genes^133^.

A new cell type can also form if cell type-defining transcription factors (or core regulatory complexes) from different cell types are co-expressed in the same cell^135^. This **cell type fusion or hybridization** can result in an increase or decrease in the number of cell types. An example is a cell type that makes a defensive gland in some beetles^178^. Cell type fusion/hybridization could be common and explain why progenitor cells often produce cells that are molecularly and morphologically distinct or why cells that are molecularly and morphologically similar originate from distinct progenitor lineages (although models like serial sister cell types^135^ can also account for some of these observations).

Early in animal evolution, new cell types emerged when cell states that originally appeared at different times evolved to occur simultaneously but in different spatial locations^136^. This **temporal-to-spatial** transition model likely still operates. For example, when developmental programs that ancestrally were sequentially activated in a lineage, co-occur^137^.

The fourth model, **terminal addition**, proposes that a new cell type can emerge through transdifferentiation (cell reprogramming) of a terminally differentiated cell type into another. For example, in zebrafish, signals from surrounding tissues prompt black pigment cells to express transcription factors that activate genes that cause these cells to break down their dark pigment and acquire crystal-like structures, becoming white pigment cells. The white and black pigment cells are morphologically and molecularly distinct and hence, considered distinct cell types ^138^.
